# The functional role of the rabbit digastric muscle during mastication

**DOI:** 10.1242/jeb.249238

**Published:** 2024-09-19

**Authors:** Roger W. P. Kissane, Karl T. Bates, Michael J. Fagan, Linjie Wang, Peter J. Watson, Graham N. Askew

**Affiliations:** ^1^ Department of Musculoskeletal and Ageing Science, University of Liverpool, The William Henry Duncan Building, Liverpool L7 8TX, UK; ^2^ School of Biomedical Sciences, University of Leeds, Leeds LS2 9JT, UK; ^3^ School of Engineering, University of Hull, Hull HU6 7RX, UK; ^4^ Structural Biomechanics, Department of Civil and Environmental Engineering, Imperial College London, London SW7 2AZ, UK; ^5^ Institute of Medical and Biological Engineering, School of Mechanical Engineering, University of Leeds, Leeds LS2 9JT, UK

**Keywords:** Muscle spindle, Muscle function, Proprioceptor, Work loop, XROMM

## Abstract

Muscle spindle abundance is highly variable in vertebrates, but the functional determinants of this variation are unclear. Recent work has shown that human leg muscles with the lowest abundance of muscle spindles primarily function to lengthen and absorb energy, while muscles with a greater spindle abundance perform active-stretch–shorten cycles with no net work, suggesting that muscle spindle abundance may be underpinned by muscle function. Compared with other mammalian muscles, the digastric muscle contains the lowest abundance of muscle spindles and, therefore, might be expected to generate substantial negative work. However, it is widely hypothesised that as a jaw-opener (anatomically) the digastric muscle would primarily function to depress the jaw, and consequently do positive work. Through a combination of X-ray reconstruction of moving morphology (XROMM), electromyography and fluoromicrometry, we characterised the 3D kinematics of the jaw and digastric muscle during feeding in rabbits. Subsequently, the work loop technique was used to simulate *in vivo* muscle behaviour *in situ*, enabling muscle force to be quantified in relation to muscle strain and hence determine the muscle's function during mastication. When functioning on either the working or balancing side, the digastric muscle generates a large amount of positive work during jaw opening, and a large amount of negative work during jaw closing, on average producing a relatively small amount of net negative work. Our data therefore further support the hypothesis that muscle spindle abundance is linked to muscle function; specifically, muscles that absorb a relatively large amount of negative work have a low spindle abundance.

## Teaser

**Summary:** Analysis of digastric muscle function during mastication provides some support for a link between spindle abundance and muscle function: muscles that absorb a relatively large amount of negative work have a low spindle abundance.

## INTRODUCTION

Skeletal muscle is functionally diverse, with its primary function often thought to be to generate force and drive movement. However, skeletal muscle also functions as a sensory organ, able to detect the position and modulate the posture of its associated body segment. Skeletal muscles comprise two unique sensory apparatus: the muscle spindle and golgi tendon organs (GTOs). Muscle spindles are intrafusal proprioceptive fibres contained within muscle fascicles, whose firing rate responds to changes in muscle length and force production (Blum et al., 2020, 2017), whereas GTOs are sensory fibres located in the muscle tendon and respond primarily to changes in force (Houk and Simon, 1967). The relative abundance of these two peripherally located sensory apparatus differ across skeletal muscle, and to date little is known about the physiological or functional determinants of this variation. Recent work modelling human walking has suggested that muscle spindle abundance may be underpinned by how a muscle functions (Kissane et al., 2022). Specifically, in the leg muscles of humans during walking, those muscles with the lowest abundance of muscle spindles primarily actively lengthen and absorb energy, while those with the greatest abundance of muscle spindles primarily function as springs (i.e. perform active-stretch–shorten cycles with no net work).

Compared with other muscles, the digastric muscle of mammals, including humans (Fig. S1) (Banks, 2006; Kissane et al., 2023), rabbits (Muhl and Kotov, 1988) and rats (Lennartsson, 1980), contains the lowest abundance of muscle spindles across the entire body (Figs S1 and S2) (Banks, 2006; Kissane et al., 2023), with Muhl and Kotov (1988) finding that 18 out of a sample of 19 rabbit digastric muscles had no muscle spindles. This low abundance of muscle spindles may be indicative of a muscle whose primary function is to act with a brake-like function, serving to dissipate energy (Kissane et al., 2022). However, it has been widely hypothesised that the digastric muscle is principally a jaw opening muscle (Meng et al., 1999; Widmalm et al., 1988), a role that would require the muscle to generate positive mechanical work to depress the jaw. In direct contrast to the digastric muscle, the temporalis muscle, a jaw closer, contains one of the highest abundances (Banks, 2006; Kissane et al., 2023) of muscle spindles (Fig. S1). Therefore, the masticatory system offers a unique opportunity to unpick the dynamic functional behaviour that may underpin muscle spindle abundance.

The digastric muscle of the rabbit is anatomically divergent from many other mammalian digastric muscles (origin – jugular process of the occipital bone; insertion – anterior ventral portion of the mandible), with its single anterior belly (Muhl et al., 1978; Tsuruyama et al., 2002) contrasting that of humans (Michna, 1989), white-tailed deer, foxes, moonrats (Turnbull, 1970), monkeys (Orsbon et al., 2018) and rats (Ciena et al., 2012) in which the muscle comprises both an anterior and an additional posterior belly. Despite these anatomical differences, the rabbit digastric muscle exhibits similar electromyography (EMG) activation timing relative to the chewing cycle to that in humans (Widmalm et al., 1988) and provides a viable model to explore the potential functional determinants of such a low muscle spindle abundance. There is a general lack of understanding of whole-muscle mechanics across muscles of the masticatory system, in part because of the difficulty in instrumenting and recording *in vivo* and *in situ* function. To date, muscle activity has been recorded (through EMG) during feeding in rabbits (Dantuma and Weijs, 1980) when operating on both the working and balancing sides, but comparable data do not exist for fibre strain during feeding. The sole attempt to record muscle strain in the rabbit digastric muscle by Muhl and Newton (1982) failed to account for feeding behaviour (i.e. working or balancing side function) and consequently their singular description of digastric muscle strain is likely to be an average of both working and balancing behaviours. Thus, no combined recording of activity and muscle strain exists, hence the mechanical function of this muscle is unknown. Therefore, in this study, we set out to quantify digastric muscle anatomy and function during feeding, and investigate how these relate to muscle spindle abundance.

It had long been thought that a small size and the presence of a relatively higher density of muscle spindles were indicative of a muscle whose function was important in fine motor control, and that muscles with longer fibres and small physiological cross-sectional area (PCSA) might function as kinesiological monitors (Barker, 1974; Peck et al., 1984; Xie et al., 2012) and consequently require a great abundance of muscle spindles. Therefore, we investigated whether muscles with greater muscle spindle abundance (e.g. medial pterygoid, superficial masseter and temporalis; Fig. S1) are architecturally aligned to function as displacement specialists (i.e. those with long muscle fibres and small PCSA), and to see how the digastric muscle with its low spindle abundance is situated among these muscles. It was hypothesised (hypothesis 1) that muscles architecturally optimised to function as displacement specialists would have the greatest muscle spindle abundance. We utilised morphospace scatterplots to present the relationship between PCSA and fibre length (Bates and Schachner, 2012; Payne et al., 2005; Wickiewicz et al., 1983) across masticatory muscles from both humans and rabbits to characterise their architectural specialisation. Our second hypothesis (hypothesis 2) was that the digastric muscle will primarily generate positive work while opening the jaw. However, with its low spindle abundance, the digastric muscle may also absorb energy and do a substantial amount of negative work during jaw closing, as hypothesised by Kissane et al. (2022). To test this hypothesis we used biplanar videography and X-ray reconstruction of moving morphology (XROMM) (Brainerd et al., 2010) to track the kinematics of the skull and mandible during feeding, as well as muscle strain trajectories (Camp et al., 2016), while simultaneously recording muscle activity using EMG. These data were used to simulate muscle function during mastication when operating on the balancing or working side, by replaying the muscle length trajectory and muscle activity pattern *in situ* using the work loop technique (Josephson, 1985). We showed that the digastric muscle when performing either working or balancing side function generates both positive and negative work during the mastication cycle, but overall generates net negative work, with the balancing side performing more net negative work than the working side. These data show that, contrary to expectation, muscles with a greater spindle abundance are not anatomically aligned with those morphologically specialised for displacement, while the digastric muscle is, despite possessing the fewest muscle spindles. Finally, our data partially support the hypothesis that muscle spindle abundance is linked to muscle function (Kissane et al., 2022, 2023): that is, low abundance is associated with muscles which actively lengthen and absorb energy, while it may not be their primary function.

## MATERIALS AND METHODS

All experimental procedures were performed in accordance with the UK Animal (Scientific Procedures) Act (1986) and approved by the Universities of Liverpool (PPL: P84984FFD) and Leeds (PPL: PA1BA29DF) Animal Welfare and Ethical Review Committees. This work conforms to the ethical requirements outlined by the journal, and is presented in accordance with the guidelines for animal work (Percie du Sert et al., 2020).

### Animals

A total of 10 New Zealand white rabbits (Envigo) (body mass 2577±181 g) were used in this study (see Figs S3 and S4 for specific animal details and experimental procedures). Animals were housed under a 12 h:12 h light:dark cycle at 21°C and had *ad libitum* access to food and water.

### Surgical procedures

Three rabbits underwent recovery surgery, conducted under aseptic conditions and under general anaesthesia. Briefly, these rabbits were premedicated with 0.35 mg kg^−1^ Medetomidine (Domitor) and 15 mg kg^−1^ ketamine (Ketaset) i.m. and intubated with a V-Gel (Docsinnovent) to maintain anaesthesia with 1–3% isoflurane (2 l min^−1^ O_2_ flow). Incisions were made at the back of the neck, across the skull and lower jaw. Three holes were drilled across the frontal and nasal bone of the skull for 1 mm tantalum beads (X-medics) to be placed and glued (Vetbond, 3M) (Fig. S3). A further three holes were drilled in the mandible diastema (across both the left and right hemimandibles) for the implantation of 1 mm tantalum beads (X-medics) and secured in place with tissue adhesive (Vetbond, 3M) (Fig. S3). EMG electrodes were tunnelled from the incision on the neck through to the mandible incision. The digastric muscle was exposed and cleared of overlying fascia, and a pair of 1 mm tantalum beads (∼6 mm apart) was implanted along a single fascicle. An offset, bipolar stainless-steel EMG electrode was then implanted into the mid-belly using a 25 gauge needle. All skin incisions were closed using 4-0 silk suture (Ethicon/Johnson & Johnson Medical, New Brunswick, NJ, USA). Animals received s.c. injections of analgesic (0.05 mg kg^−1^ buprenorphine; Buprevet^®^), anti-inflammatories (0.5 mg kg^−1^ Meloxicam, Metacam) and antibiotic (10 mg kg^−1^, 5% enrofloxacin; Baytril^®^) immediately after surgery, repeated daily for 3 days post-surgery. These three rabbits were subsequently used in the *in vivo* XROMM experiments.

### XROMM and digastric muscle strain and activity

**Fig. 1.**
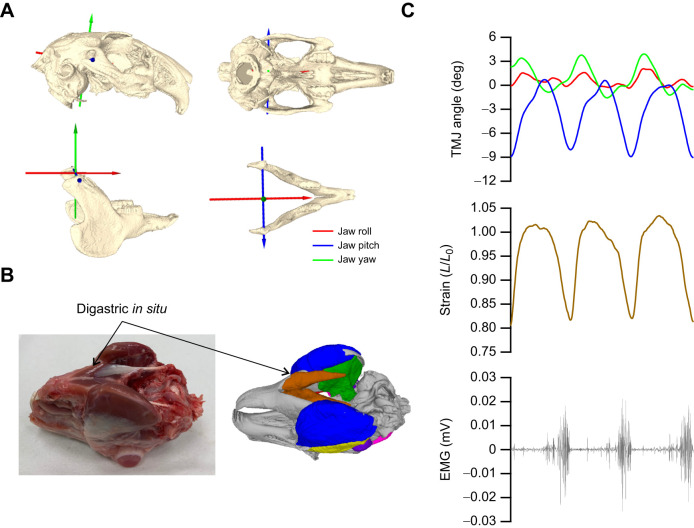
**Kinematics of rabbit mastication and anatomy of the digastric muscle.** (A) Using the X-ray reconstruction of moving morphology (XROMM) workflow, we applied joint coordinate systems to describe translations across three axes and three rotations; jaw roll, pitch and yaw. (B,C) Using implanted EMG electrodes and tantalum beads in the digastric muscle (B), we synchronously recorded muscle activity patterns, muscle length change and jaw kinematics (C). TMJ, temporomandibular joint; *L*_0_, length at which twitch force was maximal.

Rabbits were placed in a Perspex box (31 cm×52 cm×31 cm) during experimentation. Biplanar X-ray videography was completed using X-ray machines (Imaging Systems and Service, 60–70 kV, 32 mA) and recorded on two Phantom cameras (M120, Vision Research) at 1024×1024 resolution, captured at 250 Hz with shutter speed of 1/1500 during feeding on pellets (RABMA, Special Diet Services). Tantalum beads in the digastric muscle, skull and mandible were tracked in XMALab v1.5.4 (Knörlein et al., 2016) with those in the skull and mandible tracked as rigid bodies following the XROMM workflow (Knörlein et al., 2016). All rigid body translations/rotations and inter-marker distances from XMA lab were filtered using a low-pass Butterworth filter with 25 Hz cutoff frequency and exported. The joint coordinate system used here (Fig. 1A) followed previously validated conventions (Menegaz et al., 2015). Briefly, in Autodesk Maya (2019, Autodesk Inc., San Rafael, CA, USA) a neutral position *XYZ* coordinate system was set using the freely available MayaTools toolbox (https://bitbucket.org/xromm/xromm_mayatools), with the *z*-axis passing through the left and right most medial points of the mandibular condyles, the *x*-axis parallel to the occlusal plane, and the *y*-axis perpendicular to the intersect of the *z*- and *x*-axis (Fig. 1A). A total of 50 (25 working side, 25 balancing side) cycles of mastication were analysed for each of the three rabbits (*n*=150 total). Beads in the digastric muscle (Fig. 1B,C) were used to track fibre strain (Camp et al., 2016). Strain was calculated by exporting inter-bead distances from XMALab, minus resting length, and dividing this by resting inter-bead length, taken during an extended period of jaw occlusion, where no EMG activity was recorded. The precision of XROMM marker tracking was calculated as the standard deviation of the mean distance between a pair of markers within the skull and mandible (Brainerd et al., 2010; Menegaz et al., 2015). Precision values for 60 pairwise inter-marker distances measured across the three rabbits resulted in a study precision of 0.051 mm. The mean experimental tracking precision for the skull was 0.047 mm while that for the mandible was 0.054 mm. Rabbits possess a partly fused mandibular symphysis (Kraatz et al., 2021), and the use of beads implanted across the two hemimandibles to generate a single rigid body for kinematics may provide a potential source of error (Fig. S3). Here, the mean precision of beads placed in a single hemimandible was 0.040 mm while beads that spanned both sides of the mandible had a mean precision error of 0.059. Inter-marker distance of a pair of beads that span the two hemimandibles was plotted against jaw rotations across 30 masticatory cycles (Fig. S3) and suggested there to be no pattern of meaningful movement between the two hemimandibles. Therefore, these data suggest the mandible symphysis does not undergo any meaningful/detectable flexion, and subsequently that our approach assuming the mandible is a single fixed-function unit appears appropriate for the current study goals.

**Fig. 2.**
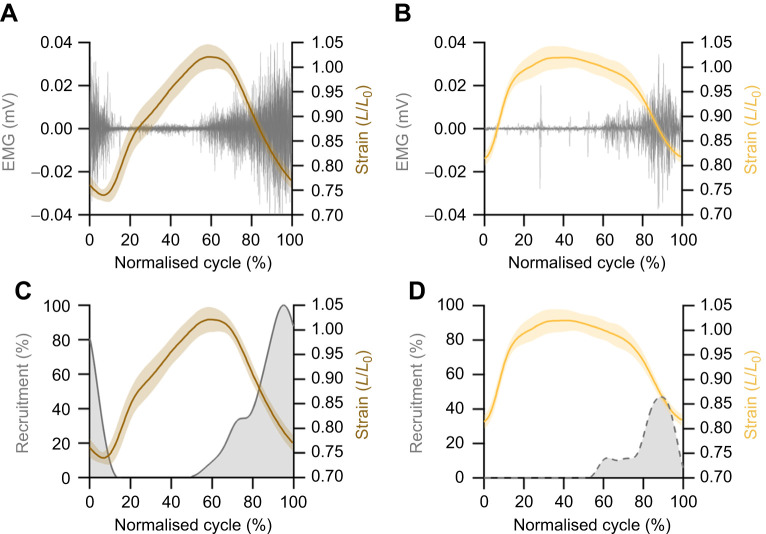
**Methodological considerations for quantification of muscle mechanics.** (A,B) *In vivo* data used to inform work loop experiments requires muscle strain amplitudes and muscle EMG to define stimulation duration on the working (A) and balancing (B) side. (C,D) Muscle recruitment patterns were replayed *in situ* on the digastric muscle to replicate the working (A,C) and balancing (B,D) side function and estimate muscle power and work. Normalised cycle (0–100%) describes the time-normalised gape cycle, where 0% corresponds to maximum gape, progressing through jaw closing, occlusion and back into reopening, with 100% corresponding with maximum gape.

EMG was recorded via an externalised connector on the back of the rabbit's neck with EMGs amplified (DAM50, ADInstruments), filtered (high pass 0.1 Hz and low pass 10 kHz) and recorded at 10 kHz using a data acquisition system (PowerLab, ADInstruments) via Labchart 8 software (ADInstruments). A square wave output signal from the Phantom Camera Controller (PCC v2.5) was used to synchronise kinematic data with EMG. The filtered EMG signal was rectified and a 30 Hz low pass filter was applied to create an activity envelope of relative activity (Fig. 2). The recruitment intensity was determined as the ratio of the integrated EMG relative to the working side, i.e. working side (100% recruitment; Fig. 2C) and balancing side (47% recruitment; Fig. 2D). The timings (onset and offset) of muscle activity relative to the strain trajectory were identified when voltages exceeded a threshold of two standard deviations of the baseline EMG signal (Roberts and Gabaldón, 2008). Following successful XROMM and EMG recordings, rabbits were euthanised with an overdose of pentobarbital.

### *In situ* muscle physiology

Muscle mechanics were measured in a second group of rabbits (*n*=4) using the EMG and muscle strain data collected and analysed from the previous *in vivo* experiments. These rabbits were anaesthetised with a s.c. injection of ketamine (Ketavet, Zoetis; 50 mg kg^−1^) and xylazine (Rompun, Bayer; 5 mg kg^−1^). Following confirmation of anaesthetic plane, an i.v. canula was implanted into the posterior facial vein to maintain sedation with ketamine and xylazine throughout the experiment. The digastric muscle was exposed via an incision running the length of the dorsal aspect of the jaw. A 3 mm hole was drilled through the mandible and a custom 3D printed frame was secured to the bone using 2.5 mm stainless steel screws. The frame was secured to a custom rig mounted on a micromanipulator, which enabled the mandible (and proximal end of the digastric muscle) to be moved relative to the ergometer along the line of action of the muscle. The distal end of the muscle was sutured to a stainless steel hoop with silk suture (3-0, LOOK Braided suture) and attached to the arm of an ergometer (305B-LR, Aurora Scientific Inc.) via a stainless steel rod. Platinum wires (0.4 mm diameter) were implanted parallel to the muscle fibres using a 25 gauge needle and sutured into place (Kissane and Askew, 2024), and generated isometric tetanic forces comparable to those obtained via nerve cuff stimulation (Anapol et al., 1987; Muhl et al., 1978) (Fig. S4). The rabbit's body temperature was maintained at 37°C (Animal Temperature Controller 2000, WPI) and the muscle was regularly irrigated with warmed (37°C) saline. Following completion of the experiment, the rabbit was humanely euthanised with an overdose of pentobarbital.

### Muscle work loop experiments

The digastric muscle was subjected to a series of supramaximal isometric twitches (0.2 ms pulse) and muscle length was incrementally increased by 1 mm to find the length at which twitch force was maximal (defined as *L*_0_). Without direct measures of *in vivo* whole digastric muscle length change, it is difficult to directly relate muscle resting length with the optimum muscle length. Lengthening a muscle on the descending limb of the length–force relationship can give rise to instability (due to longer, weaker sarcomeres being susceptible to further lengthening, rendering them even weaker; Morgan, 1990) and is unlikely to be physiological. Therefore, cyclical length changes were imposed on the muscle such that the maximum length attained was on the plateau of the length–force relationship, and muscle operated on the ascending limb and plateau of the length–force relationship (Fig. S5).

The work loop technique (Josephson, 1985) was used to quantify the instantaneous power output of the digastric muscle by subjecting the muscle to the strain trajectory recorded *in vivo* relative to *L*_0_ (Fig. 2A,B). Traditionally, supramaximal, square wave muscle activation has been used to activate muscles during work loop protocols (Askew and Marsh, 1997, 2001). However, with modification to the Aurora Scientific stimulator (model 701C, Aurora Scientific Inc.), it is possible to deliver time varied voltage/current outputs that replicate EMG activation patterns (Bukovec et al., 2020). Here, we utilised this modified approach to replay muscle recruitment patterns of the digastric muscle that better reflect *in vivo* levels of recruitment and, therefore, function (Fig. 2C,D) (Charles et al., 2024). Using an incremental current–recruitment curve of isometric twitches, we determined the current required to maximally activate the digastric muscle, and the minimum current required to evoke a force response. The corresponding sigmoidal current–recruitment curve was used to determine levels of current required to maximally activate the muscle (working side=100% activation; Fig. 2C), and the subsequent recruitment levels associated with the balancing side EMG (47%; Fig. 2D). The aforementioned envelope of relative activity determined from the analysis of the EMG signal served to control the current delivered to the muscle in order to match the recruitment intensity *in vivo*. Muscles were subjected to 5 repeated cycles that simulated (0.2 ms pulse width, 200 Hz) the *in vivo* strain trajectory, with an initial passive work loop followed by four active work loops (stimuli delivered at 200 Hz, the fusion frequency of the muscle during an isometric tetanus). Muscles were subjected to the two different work loop conditions (i.e. working side recruitment and balancing side recruitment), which were presented to the muscle in a randomised order.

### Digastric muscle architecture

The architecture of the digastric muscle was measured in four rabbits. Firstly, muscle fibre pennation angle was measured relative to the line of action of the muscle *in vivo*. Subsequently, the digastric muscles were dissected free, weighed, and then fixed in 4% neutrally buffered formalin for 24 h at approximately resting muscle length. Following fixation, muscles were bathed in 30% nitric acid to free muscle fibres from their connective tissue. Digestion was ceased by transferring the fibre bundles into a 50:50 glycerol:dH_2_O solution. Twenty fibres were measured from each muscle and mean muscle fibre length was determined relative to resting muscle length. PCSA (mm^2^) was calculated for the digastric muscle as described in Eqn 1:
(1)

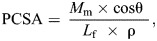
where *M*_m_ is muscle mass (in g), cosθ is the pennation angle of the muscle fibres relative to the line of action of the muscle, *L*_f_ is muscle fibre length (in mm) and ρ is muscle density (0.001056 g mm^3^) (Charles et al., 2022; Martin et al., 2020; Mendez et al., 1960). We utilised morphospace scatterplots to present the relationship between PCSA and fibre length (Allen et al., 2010; Bates and Schachner, 2012; Charles et al., 2022; Kissane et al., 2022, 2023; Schachner et al., 2024) to compare the digastric muscle architecture with that of the major jaw closing muscles taken from Watson et al. (2014). Muscles with relatively long fibre lengths and low PCSA were classed as ‘displacement specialised’, those with relatively short fibre lengths and high PCSA as ‘force specialised’, and those with relatively long fibre lengths and high PCSA as ‘power specialised’ (Allen et al., 2010; Charles et al., 2022; Kissane et al., 2022, 2023).

### Statistics

Statistical differences in muscle length trajectory were quantified using one-dimensional statistical parametric mapping (1D SPM) (Pataky, 2012) in Matlab (R2018a, MathWorks). All remaining statistics were completed using SPSS 28 (28.0.1.1, IBM). EMG timings, muscle power, and positive and negative work between working and balancing side function were compared using an independent *t*-test, with the threshold for statistical significance set to *P*<0.05. All data processing and figures were plotted using Igor Pro 8 (v8.0.4.2, Wavemetrics) and are presented as means±s.d.

## RESULTS

### The functional anatomy of the digastric muscle

**Fig. 3.**
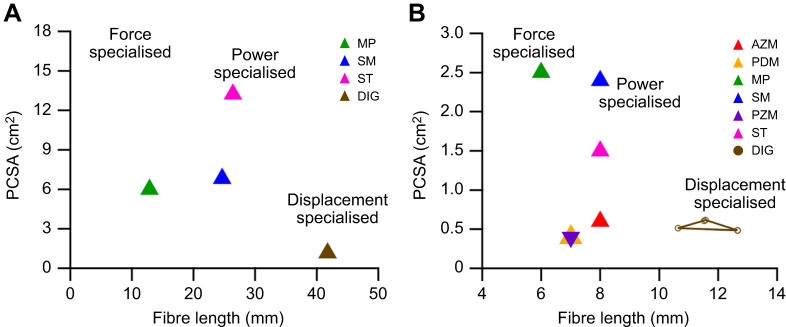
**Morphospace plot for the jaw muscles in humans and rabbits.** (A) Fibre length and physiological cross-sectional area (PCSA) for human jaw muscles taken from Kissane et al. (2023). (B) Fibre length and PCSA for the rabbit masticatory muscles; here, we measured the digastric (DIG) muscle (circles, *n*=4), while data for the jaw closing muscles (triangles) were taken from Watson et al. (2014): AZM, anterior zygomaticomandibularis; PDM, posterior deep masseter; PT, medial pterygoid; SM, superficial masseter; PZM, posterior zygomaticomandibularis; and ST, superficial temporalis. The small PCSA and long fibre length of the DIG muscle suggest an architecture optimised to function as a displacement specialist.

Jaw muscle architecture was collated for humans (Kissane et al., 2023; Van Eijden et al., 1997) and rabbits (Watson et al., 2014). In humans, the functional morphospace plot (Fig. 3A) shows that across all jaw musculature, the digastric muscle is the most architecturally optimised to function as a displacement specialist, with a relatively low PCSA (1.16±0.3 cm^2^) and relatively long mean fibre length (41.8±5.3 mm). In the rabbit, the mean digastric muscle fibre length was 11.59±0.83 mm (Fig. 3B) relative to the whole muscle length of 34.75±1.17 mm, equating to an average fibre to muscle length ratio of 0.33. The digastric muscle fibres have a pennation angle of 9.81±1.52 deg relative to the line of action of the muscle; consequently, the calculated muscle PCSA was 0.556±0.067 cm^2^. Similar to humans, the morphospace plots of PCSA and fibre length for the rabbit suggest that the digastric muscle is, architecturally, a displacement specialist compared with the major jaw closing muscles (Fig. 3B). These data across the masticatory system of rabbits and humans do not support the hypothesis that muscles optimised for displacement function possess a higher spindle abundance (Fig. S6; hypothesis 1).

### *In vivo* muscle length trajectory and activity patterns of the digastric muscle during feeding

**Fig. 4.**
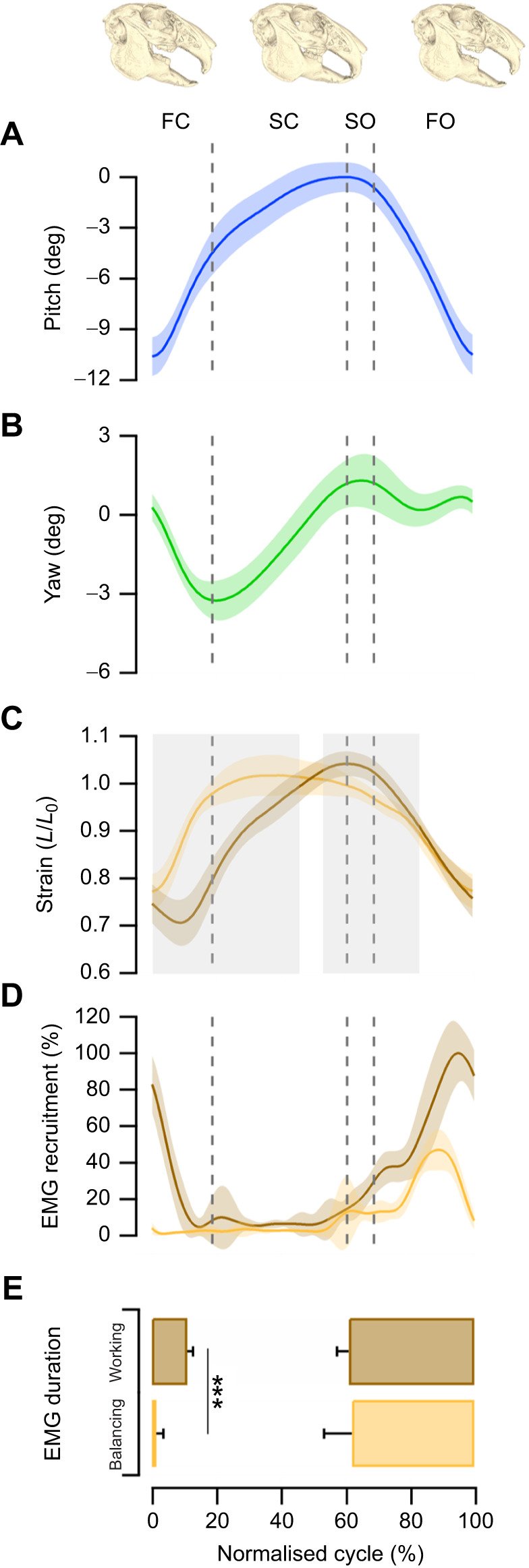
**Digastric function during feeding.** (A,B) Time-normalised data for the pitch (A) and yaw (B) of the jaw starting at maximum gape, and progressing through occlusion and re-opening (*n*=150 cycles). (C) The muscle strain experienced by the digastric muscle is dependent on whether it functions on the working (brown) or balancing (orange) side of the jaw. The grey shaded area highlights significantly different (*P*<0.001) strain amplitudes by 1D SPM. (D,E) Muscle EMG recruitment curves (D) and initiation and cessation of muscle EMG for the working and balancing side digastric muscle (E) show that when functioning on the working side, it is active long into the fast-closing phase. Data are presented as means±s.d., ***P<0.001. Dashed lines correspond to transitional phases: FC, fast closing; SC, slow closing; SO, slow opening; FO, fast opening.

Masticatory cycle kinematic data are presented as a normalised cycle (0–100%) where 0% corresponds to peak gape, progressing through jaw closing, occlusion and back to reopening, with 100% corresponding with maximum gape (Bramble and Wake, 1985; Orsbon et al., 2020). The masticatory cycle of mammals is commonly broken down into four constituent phases (Fig. 4). First a fast closing phase where the food bolus is prepared and placed into position ready for the forceful biting. This is followed by the slow closing phase (or power stroke), which involves crushing of the food bolus. Following complete occlusion, the jaw then undergoes a slow opening phase and finally a fast opening phase. During pellet feeding in the rabbits, the four phases occurred during the time-normalised masticatory cycle as: fast closing 0–18.66%, slow closing 18.66–60.51%, slow opening 60.51–68.84% and fast opening 68.84–100% (Fig. 4). The digastric muscle was active throughout slow and fast opening of the jaw, and parts of fast closing (Fig. 4). The digastric muscle presents with significantly distinct muscle length trajectories when functioning on the working side compared with the balancing (1D SPM, significantly different portions of the masticatory cycle: 0–45.5%, 53–82.5%, *P*<0.001; Fig. 4C). Accordingly, the digastric muscle when undergoing balancing side length trajectories has a strain of 23±5% while the working side has a strain of 32±8%.

Muscle EMG onset relative to the masticatory cycle (maximum gape to maximum gape) did not significantly differ between the working and balancing side functions (61.09±3.65% gape cycle versus 62.08±8.65% gape cycle, *t*_92_=−0.724, *P*=0.471; Fig. 4D,E). However, the relative EMG offset was dependent on whether the muscle was functioning on the working side or balancing side (10.99±1.86% gape cycle versus 1.45±2.23% gape cycle, *t*_92_=22.551, *P*<0.001; Fig. 4D,E). The working side activation coincided with lateral displacement of the jaw during feeding (Fig. 4B,D,E).

### The mechanical properties of the digastric muscle during isometric and simulated *in vivo* contractions

**Fig. 5.**
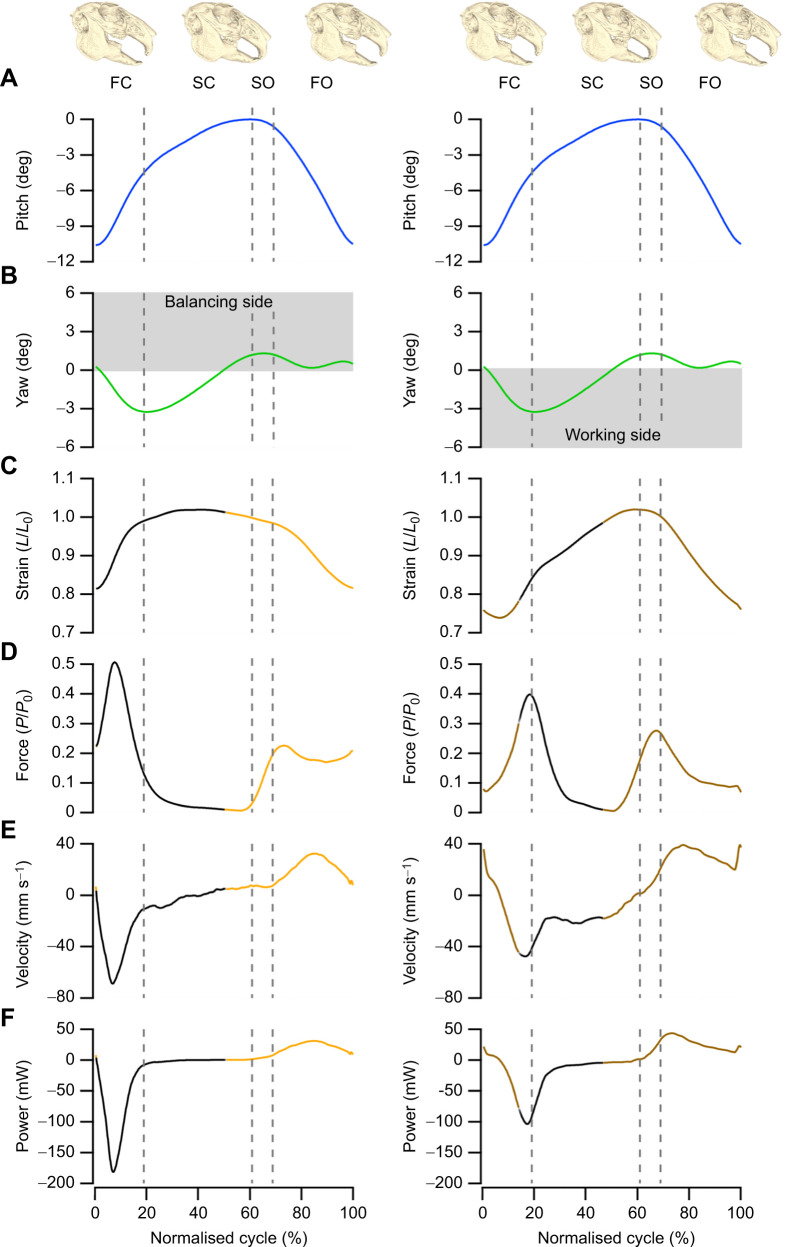
**Example from one individual of the mechanical properties of the digastric muscle during feeding.** (A,B) Time-normalised data for the pitch (A) and yaw (B) of the jaw starting at maximum gape, progressing through occlusion, and re-opening. (C,D) The fibre strain replayed onto the digastric muscle (C) and the subsequent force generated by the muscle (D) highlight the largest forces are eccentrically generated during jaw opening on both the working and balancing side. (E) The different strain trajectories across the two feeding behaviours undergo distinct muscle fibre velocities, with the balancing side experiencing a greater muscle lengthening velocity than that of the working side. (F) Consequently, the greater force and velocity experienced by the balancing side correspond to a greater amount of negative power generated by the balancing side during jaw opening. However, the working side generates more positive work during jaw opening. Balancing side data (left column) are presented in orange (active stimulation) and in black (inactive) and working side data (right column) are presented in brown (active stimulation) and in black (inactive). Dashed lines correspond to transitional phases: FC, fast closing; SC, slow closing; SO, slow opening; FO, fast opening. *P*_0_, maximum isometric tetanic force.

**Fig. 6.**
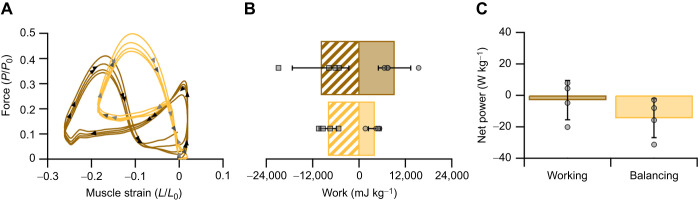
**Digastric work loops derived from *in vivo* recordings.** (A) Muscle strain trajectories and recruitment replicating digastric muscle functioning as either the balancing (orange) or working (brown) side resulted in distinct work loop shapes. (B) The net work generated by the muscle (*n*=4) was not significantly different as determined by independent *t*-test, with the working side (brown) generating similar positive and negative work to that of the balancing side (orange). (C) The different functional behaviours of the digastric muscle did not generate significantly different power, but principally generated negative power. A representative example is presented in A and data in B and C are presented as means±s.d.

The digastric muscle had an isometric twitch rise time of 36.25±0.6 ms and a twitch half-relaxation time of 31.0±0.3 ms. When fully tetanised, the digastric muscle generated 132.9±15.1 kN m^−2^ of isometric stress at *L*_0_. The twitch to tetanus ratio for the digastric muscle was 0.43±0.08. Replayed muscle length trajectories and activity patterns onto the digastric muscle generated distinct force and power profiles for the balancing and working side kinematics (Fig. 5). The digastric muscle was actively shortened during jaw opening and generated force and positive power during this phase of the mastication cycle when operating on both the working and balancing side (Figs 4 and 5). Muscle recruitment was 53% higher when the muscle operated on the working side (Fig. 4), resulting in higher forces and greater work generation, compared with when the muscle operated on the balancing side (Fig. 5). Muscle activity continued into the fast closing phase of the mastication cycle whilst the digastric muscle undergoes rapid lengthening, resulting in a large peak in force and a corresponding large, negative instantaneous power; force decreased following cessation of muscle activity during the slow closing phase of the mastication cycle, with minimal power generation (Fig. 5). Muscle work loops (Fig. 6A) highlight the relatively high positive work generated in the initial shortening phase of the cycle (anti-clockwise work loop) followed by the large negative work (predominantly clockwise work loop) as the muscle was stretched; similar work loop trajectories were found when the digastric muscle operated on both the working and balancing side. Over the mastication cycle, the muscle generated similar amounts of negative (−10,027±7325 mJ kg^−1^ versus −8166±2258 mJ kg^−1^, *t*_6_=−0.485, *P*=0.322; Fig. 6B) and positive work (9124±4174 mJ kg^−1^ versus 4030±1633 mJ kg^−1^, *t*_6_=2.273, *P*=0.063; Fig. 6B) when operating on both the working and balancing side. Interestingly, the magnitude of positive and negative work did not differ for the working side (9124±4175 mJ kg^−1^ versus 10,027±7325 mJ kg^−1^, *t*_6_=−0.214, *P*=0.419), but were significantly different for the balancing side (4030±1633 mJ kg^−1^ versus 8166±2258 mJ kg^−1^, *t*_6_=−2.968, *P*=0.013), respectively. This resulted in comparable levels of net work when operating on the working and balancing side (−903±3405 mJ kg^−1^ versus −4136±3478 mJ kg^−1^, *t*_6_=1.329, *P*=0.232). Consequently net muscle power was negative, and not significantly different, during the two modes of action (*t*_6_=1.295, *P*=0.122; Fig. 6C), when functioning as a working (−3.04±12.51 W kg^−1^) or balancing (−14.45±12.43 W kg^−1^) side muscle.

## DISCUSSION

The neuromuscular innervation (Tsuruyama et al., 2002), phenotype (Korfage et al., 2006) and temporal activation (Tsuruyama et al., 2002) of the rabbit digastric muscle have been relatively well characterised in the literature. However, limited muscle mechanics data exist for the rabbit digastric muscle, with only the force–length (Muhl et al., 1978) and force–velocity relationship (Anapol et al., 1987) having been characterised in the whole muscle–tendon unit. The muscle length trajectory and force generation during feeding, and consequently the muscle's mechanical function, have never been characterised. Here, we used a modified work loop approach (Josephson, 1985) to replay representative patterns of *in vivo* muscle length change and recruitment (Bukovec et al., 2020; Charles et al., 2024) to investigate the mechanical function of the digastric muscle during feeding. We show that the digastric muscle architecture is consistent with it operating as a displacement specialist and does not support our first hypothesis that muscles optimised to undergo large length excursions require greater muscle spindle abundance (Barker, 1974; Peck et al., 1984). Additionally, we have shown that the digastric muscle undergoes distinct patterns of muscle activity and length trajectories depending on whether the muscle is operating on the working or on the balancing side during feeding, which correspond with distinct force and instantaneous power profiles. Fundamentally, these data add further support to our second hypothesis, suggesting that a low muscle spindle abundance is associated with muscles that generate negative work. Here, we show that the digastric muscle functions predominantly as an energy absorber (the muscle generates net negative work) during jaw closing, while the muscle also generates relatively high positive work during jaw opening.

The muscle architecture and morphospace plots show the digastric muscle has the longest fibres and the smallest PCSA of all the jaw musculature across both humans and rabbits and suggest that the digastric muscle is architecturally optimised to undergo large length excursions (Charles et al., 2022). Supporting this prediction based on muscle architecture, our *in vivo* recordings of muscle length show that the digastric muscle undergoes relatively large strains when operating on both the working (32%) and balancing (23.4%) sides during feeding. These peak-to-peak strains are larger than many recorded in vertebrate muscles involved in walking [∼25% in the gastrocnemius muscle of goats (Lee et al., 2013) and ∼20% in the medial gastrocnemius muscle and ∼8% in the soleus and plantaris muscles of the rat (Hodson-Tole and Wakeling, 2010)], swimming [15% in the red muscle of scup (Coughlin et al., 1996) and 20% in the superficial white muscle of carp (Wakeling and Johnston, 1999)] and flight [21–23% in the quail pectoralis muscle (Askew and Marsh, 2001), on average 19% in the pigeon pectoralis muscle (Biewener et al., 1998), and 29% in the pectoralis muscle of the mallard (Williamson et al., 2001)]. Here, we provide some of the first masticatory muscle strain data records, with only architectural gear ratios having been reported previously in the temporalis of the capuchin (Laird et al., 2020). These data highlight that, contrary to previously held beliefs (Barker, 1974; Peck et al., 1984), muscles with greater muscle spindle abundance (e.g. superficial masseter, medial pterygoid and temporalis muscles) are not anatomically optimised to function as displacement specialists.

When operating on the working side, the digastric muscle was active predominantly during shortening, resulting in force generation during both the slow- and fast-opening phase of the masticatory cycle, generating positive work and instantaneous power to open the jaw. On the balancing side, the muscle underwent comparable shortening velocity but over a shorter amplitude (Fig. 5E) and with a lower EMG recruitment. Consequently, our *in situ* preparation predicts the digastric muscle when functioning on the working side generates greater force, instantaneous power and positive work during jaw opening than that of the balancing side. The activity of the digastric muscle continues into jaw closing, with the highest forces being generated during this phase of the masticatory cycle. Thus, the digastric muscle appears to oppose the jaw closing forces of the masseter muscles, and simultaneously functions to stabilize the jaw during the rotational yaw of the mandible, against the pterygoid (Dantuma and Weijs, 1980; Weijs and Muhl, 1987). The role in jaw stabilisation appears to be important when the muscle operates on either the working or balancing side, given that the magnitude of the energy absorbed is similar. Previous recordings in rabbits have largely focused on the vertical displacement of the jaw, and subsequently ignored jaw yaw (i.e. medial lateral rotations). These previous studies have shown the digastric muscle to be active during jaw opening (Lund and Rossignol, 1981; Tsuruyama et al., 2002), with some suggestion of activity into the fast closing phase (Schwartz et al., 1989). Data from Schwartz and colleagues (1989) qualitatively suggest that the digastric muscle is active into parts of jaw closing, and potentially involved in lateral displacement of the jaw; however, this is not explicitly quantified and, based on EMG activity alone, not force.

As a whole muscle–tendon unit, it has been shown that the rabbit digastric tendon may not undergo length change during feeding (Muhl and Newton, 1982), though the authors of that study acknowledge that the sensitivity of the approach used to collect these data may be inadequate, and further work is needed to confirm this. However, at present, the tendon appears to function as a stiff linkage between the muscle and the skeleton that serves simply to transmit the forces developed by the muscle, providing much better control of jaw position than if the tendon was more compliant. Our data here provide a qualitative insight into the dynamic role of the digastric muscle during feeding (Ahn and Full, 2002). The digastric muscle has a relatively small pennation angle at resting length (approximately 10 deg). Under passive lengthening of the digastric muscle, Muhl (1982) showed that the pennation angle may increase to up to 27 deg in the posterior fibres and 20 deg in the anterior fibres. To date, no comprehensive assessment of muscle gearing has been undertaken in the digastric muscle during active feeding, in part because of the complex nature of those experiments (Ross et al., 2024). It is therefore not known to what extent (if any) muscle pennation may be changing under active cyclical loading in this muscle. Here, we have measured muscle fascicle length change to replay *in situ*, assuming a comparable level of muscle gearing is taking place in our *in situ* experiments to that *in vivo*. There is evidence to suggest that changes in pennation angle during active cyclical contractions may have a negligible contribution to the force dynamics of a muscle (Lieber, 2022). Therefore, our data on the proportions of positive and negative net work for the digastric muscle are probably closely representative of how the digastric muscle functions during mastication.

The digastric muscle is interchangeably grouped with either the jaw musculature (Banks, 2006) for its primary function as a jaw opener or the hyoid muscles (Mayerl et al., 2021a) as it also functions during swallowing. Interestingly, there is a strong relationship between functional groups of muscles and their composition of muscle spindles (Banks, 2006; Kissane et al., 2023). Muscles of the jaw (e.g. temporalis, masseter and pterygoid muscles) have some of the greatest muscle spindle abundance across the body, while the hyoid musculature (e.g. stylohyoideus, geniohyoideus, thyrohyoid and sternohyoid) has the lowest abundance (Banks, 2006; Kissane et al., 2023). We have recently suggested that muscle spindle abundance is linked to muscle function *in vivo*, with muscles containing a greater abundance of spindles primarily functioning as springs (i.e. performing active-stretch–shorten cycles with no net work), and those with less abundance functioning to absorb energy (i.e. producing negative work; Kissane et al., 2022, 2023). The initial activation of the digastric muscle corresponds with the initiation of the slow opening phase, and subsequently the large portion of muscle shortening. This means that during jaw opening the digastric muscle functions as a motor, generating positive work. However, the maintenance of activation into jaw closing as the muscle is lengthening results in large amounts of negative work, and the muscle additionally functioning to absorb energy. Despite generating similar amounts of positive and negative work, the muscle is unlikely to operate as a spring as the generation of positive work is preceded by a period when the muscle is inactive and generates no force. Our data here in the rabbit partially support our second hypothesis, with the digastric muscle generating negative work to stabilise the mandible during jaw closing, while the muscle also generates a significant proportion of positive work during jaw opening. Data from the pig hyoid musculature show the geniohyoid, thyrohyoid and omohyoid muscles are similarly active during lengthening (Mayerl et al., 2021a,b), which could suggest they do large amounts of negative work, but as has been shown in the rabbit digastric muscle, the temporal delay in force generation relative to muscle activity makes it likely that these muscles also generate both negative and positive work. Inferring function from presumed timing of force generation based on recruitment, as opposed to measuring forces (as we report in the rabbit digastric muscle), is beset with uncertainty given that the electromechanical delay for relaxation is variable (Roberts and Gabaldon, 2008). The masticatory system offers a unique opportunity to explore the dynamic behaviour of muscles contained within the same function group (Ahn and Full, 2002), in addition to the potential functional underpinning of muscle spindle abundance.

Our data provide a unique insight into the possible mechanical function of the digastric muscle during feeding in the rabbit. We have shown that the muscle undergoes distinct length trajectories when feeding, and that it is capable of dynamically modifying activation patterns to modulate force production when functioning on either the working or balancing side of the jaw. These data emphasise the versatility of muscle function within a single muscle and highlight the uniqueness of mechanical function within the masticatory system. Critically, we provide some support to the hypothesis that muscle spindle abundance is underpinned by muscle function. Finally, these direct measures of muscle mechanical performance may be used to validate musculoskeletal models of mastication (Watson et al., 2014), which may in turn be used to probe motor control strategies and physiological determinants of muscle spindle abundance.

## Supplementary Material

10.1242/jexbio.249238_sup1 Supplementary information

## References

[JEB249238C1] Ahn, A. N. and Full, R. J. (2002). A motor and a brake: two leg extensor muscles acting at the same joint manage energy differently in a running insect. *J. Exp. Biol.* 205, 379-389. 10.1242/jeb.205.3.379 11854374

[JEB249238C2] Allen, V., Elsey, R. M., Jones, N., Wright, J. and Hutchinson, J. R. (2010). Functional specialization and ontogenetic scaling of limb anatomy in *Alligator mississippiensis*. *J. Anat.* 216, 423-445. 10.1111/j.1469-7580.2009.01202.x 20148991 PMC2849521

[JEB249238C3] Anapol, F., Muhl, Z. and Fuller, J. (1987). The force-velocity relation of the rabbit digastric muscle. *Arch. Oral Biol.* 32, 93-99. 10.1016/0003-9969(87)90051-3 3478015

[JEB249238C4] Askew, G. N. and Marsh, R. L. (1997). The effects of length trajectory on the mechanical power output of mouse skeletal muscles. *J. Exp. Biol.* 200, 3119-3131. 10.1242/jeb.200.24.3119 9364020

[JEB249238C5] Askew, G. N. and Marsh, R. L. (2001). The mechanical power output of the pectoralis muscle of blue-breasted quail (*Coturnix chinensis*): the in vivo length cycle and its implications for muscle performance. *J. Exp. Biol.* 204, 3587-3600. 10.1242/jeb.204.21.3587 11719526

[JEB249238C6] Banks, R. (2006). An allometric analysis of the number of muscle spindles in mammalian skeletal muscles. *J. Anat.* 208, 753-768. 10.1111/j.1469-7580.2006.00558.x 16761976 PMC2100235

[JEB249238C7] Barker, D. (1974). The morphology of muscle receptors. In *Muscle Receptors*, pp. 1-190. Springer.

[JEB249238C8] Bates, K. T. and Schachner, E. R. (2012). Disparity and convergence in bipedal archosaur locomotion. *J. R Soc. Interface* 9, 1339-1353. 10.1098/rsif.2011.0687 22112652 PMC3350733

[JEB249238C9] Biewener, A. A., Corning, W. R. and Tobalske, B. W. (1998). In vivo pectoralis muscle force–length behavior during level flight in pigeons (*Columba livia*). *J. Exp. Biol.* 201, 3293-3307. 10.1242/jeb.201.24.3293 9817827

[JEB249238C10] Blum, K. P., Lamotte D'Incamps, B., Zytnicki, D. and Ting, L. H. (2017). Force encoding in muscle spindles during stretch of passive muscle. *PLoS Comput. Biol.* 13, e1005767. 10.1371/journal.pcbi.1005767 28945740 PMC5634630

[JEB249238C11] Blum, K. P., Campbell, K. S., Horslen, B. C., Nardelli, P., Housley, S. N., Cope, T. C. and Ting, L. H. (2020). Diverse and complex muscle spindle afferent firing properties emerge from multiscale muscle mechanics. *Elife* 9, e55177. 10.7554/eLife.55177 33370235 PMC7769569

[JEB249238C12] Brainerd, E. L., Baier, D. B., Gatesy, S. M., Hedrick, T. L., Metzger, K. A., Gilbert, S. L. and Crisco, J. J. (2010). X-ray reconstruction of moving morphology (XROMM): precision, accuracy and applications in comparative biomechanics research. *J. Exp. Zool. Part A: Ecol. Genetics Physiol.* 313A, 262-279. 10.1002/jez.589 20095029

[JEB249238C13] Bramble, D. M. and Wake, D. B. (1985). Chapter 13. Feeding mechanisms of lower tetrapods. In *Functional Vertebrate Morphology*, pp.230-261. Harvard University Press.

[JEB249238C14] Bukovec, K. E., Hu, X., Borkowski, M., Jeffery, D., Blemker, S. S. and Grange, R. W. (2020). A novel ex vivo protocol to mimic human walking gait: implications for Duchenne muscular dystrophy. *J. Appl. Physiol.* 129, 779-791. 10.1152/japplphysiol.00002.2020 32881620 PMC7654698

[JEB249238C15] Camp, A. L., Astley, H. C., Horner, A. M., Roberts, T. J. and Brainerd, E. L. (2016). Fluoromicrometry: a method for measuring muscle length dynamics with biplanar videofluoroscopy. *J. Exp. Zool. Part A: Ecol. Genetics Physiol.* 325, 399-408. 10.1002/jez.2031 27488475

[JEB249238C16] Charles, J., Kissane, R., Hoehfurtner, T. and Bates, K. T. (2022). From fibre to function: are we accurately representing muscle architecture and performance? *Biol. Rev.* 97, 1640-1676. 10.1111/brv.12856 35388613 PMC9540431

[JEB249238C17] Charles, J. P., Kissane, R. W. and Askew, G. N. (2024). The impacts of muscle-specific force-velocity properties on predictions of mouse muscle function during locomotion. *Front. Bioeng. Biotechnol.* 12, 1436004. 10.3389/fbioe.2024.1436004 39108597 PMC11300213

[JEB249238C18] Ciena, A. P., De Almeida, S. R. Y., Dias, F. J., De Sousa Bolina, C., Issa, J. P. M., Iyomasa, M. M., Ogawa, K. and Watanabe, I.-S. (2012). Fine structure of myotendinous junction between the anterior belly of the digastric muscle and intermediate tendon in adults rats. *Micron* 43, 258-262. 10.1016/j.micron.2011.08.009 21967838

[JEB249238C19] Coughlin, D. J., Valdes, L. and Rome, L. C. (1996). Muscle length changes during swimming in scup: sonomicrometry verifies the anatomical high-speed cine technique. *J. Exp. Biol.* 199, 459-463. 10.1242/jeb.199.2.459 8930001

[JEB249238C20] Dantuma, R. and Weijs, W. (1980). Functional anatomy of the masticatory apparatus in the rabbit (Oryctolagus cuniculus L.). *Neth. J. Zool.* 31, 99-147. 10.1163/002829680X00212

[JEB249238C21] Hodson-Tole, E. F. and Wakeling, J. (2010). The influence of strain and activation on the locomotor function of rat ankle extensor muscles. *J. Exp. Biol.* 213, 318-330. 10.1242/jeb.031872 20038667

[JEB249238C22] Houk, J. and Simon, W. (1967). Responses of Golgi tendon organs to forces applied to muscle tendon. *J. Neurophysiol.* 30, 1466-1481. 10.1152/jn.1967.30.6.1466 6066449

[JEB249238C23] Josephson, R. K. (1985). Mechanical power output from striated muscle during cyclic contraction. *J. Exp. Biol.* 114, 493-512. 10.1242/jeb.114.1.493

[JEB249238C24] Kissane, R. W. P. and Askew, G. N. (2024). Conserved mammalian muscle mechanics during eccentric contractions. *J. Physiol.* 602, 1105-1126. 10.1113/JP285549 38400808

[JEB249238C25] Kissane, R. W., Charles, J. P., Banks, R. W. and Bates, K. T. (2022). Skeletal muscle function underpins muscle spindle abundance. *Proc. R. Soc. B* 289, 20220622. 10.1098/rspb.2022.0622 PMC9156921 35642368

[JEB249238C26] Kissane, R. W., Charles, J. P., Banks, R. W. and Bates, K. T. (2023). The association between muscle architecture and muscle spindle abundance. *Sci. Rep.* 13, 2830. 10.1038/s41598-023-30044-w 36806712 PMC9938265

[JEB249238C27] Knörlein, B. J., Baier, D. B., Gatesy, S. M., Laurence-Chasen, J. and Brainerd, E. L. (2016). Validation of XMALab software for marker-based XROMM. *J. Exp. Biol.* 219, 3701-3711. 10.1242/jeb.145383 27655556

[JEB249238C28] Korfage, J., Van Wessel, T., Langenbach, G., Ay, F. and Van Eijden, T. (2006). Postnatal transitions in myosin heavy chain isoforms of the rabbit superficial masseter and digastric muscle. *J. Anat.* 208, 743-751. 10.1111/j.1469-7580.2006.00562.x 16761975 PMC2100230

[JEB249238C29] Kraatz, B., Belabbas, R., Fostowicz-Frelik, Ł., Ge, D.-Y., Kuznetsov, A. N., Lang, M. M., López-Torres, S., Mohammadi, Z., Racicot, R. A., Ravosa, M. J. et al. (2021). Lagomorpha as a model morphological system. *Front. Ecol. Evol.* 9, 636402. 10.3389/fevo.2021.636402

[JEB249238C30] Laird, M. F., Granatosky, M. C., Taylor, A. B. and Ross, C. F. (2020). Muscle architecture dynamics modulate performance of the superficial anterior temporalis muscle during chewing in capuchins. *Sci. Rep.* 10, 6410. 10.1038/s41598-020-63376-y 32286442 PMC7156371

[JEB249238C31] Lee, S. S., De Boef Miara, M., Arnold, A. S., Biewener, A. A. and Wakeling, J. M. (2013). Recruitment of faster motor units is associated with greater rates of fascicle strain and rapid changes in muscle force during locomotion. *J. Exp. Biol.* 216, 198-207. 10.1242/jeb.072637 22972893 PMC3597201

[JEB249238C32] Lennartsson, B. (1980). Number and distribution of muscle spindles in the masticatory muscles of the rat. *J. Anat.* 130, 279. 6447135 PMC1233132

[JEB249238C33] Lieber, R. L. (2022). Can we just forget about pennation angle? *J. Biomech.* 132, 110954. 10.1016/j.jbiomech.2022.110954 35074689

[JEB249238C34] Lund, J. and Rossignol, S. (1981). Modulation of the amplitude of the digastric jaw opening reflex during the masticatory cycle. *Neuroscience* 6, 95-98. 10.1016/0306-4522(81)90247-5 7219709

[JEB249238C35] Martin, M. L., Travouillon, K. J., Fleming, P. A. and Warburton, N. M. (2020). Review of the methods used for calculating physiological cross–sectional area (PCSA) for ecological questions. *J. Morphol.* 281, 778-789. 10.1002/jmor.21139 32374505

[JEB249238C36] Mayerl, C., Steer, K., Chava, A., Bond, L., Edmonds, C., Gould, F., Stricklen, B., Hieronymous, T. and German, R. (2021a). The contractile patterns, anatomy and physiology of the hyoid musculature change longitudinally through infancy. *Proc. R. Soc. B* 288, 20210052. 10.1098/rspb.2021.0052 PMC7944089 33715426

[JEB249238C37] Mayerl, C. J., Steer, K. E., Chava, A. M., Bond, L. E., Edmonds, C. E., Gould, F. D., Hieronymous, T. L., Vinyard, C. J. and German, R. Z. (2021b). Anatomical and physiological variation of the hyoid musculature during swallowing in infant pigs. *J. Exp. Biol.* 224, jeb243075. 10.1242/jeb.243075 34734633 PMC10659033

[JEB249238C38] Mendez, J., Keys, A., Anderson, J. and Grande, F. (1960). Density of fat and bone mineral of the mammalian body. *Metabolism* 9, 472-477.

[JEB249238C39] Menegaz, R. A., Baier, D. B., Metzger, K. A., Herring, S. W. and Brainerd, E. L. (2015). XROMM analysis of tooth occlusion and temporomandibular joint kinematics during feeding in juvenile miniature pigs. *J. Exp. Biol.* 218, 2573-2584. 10.1242/jeb.119438 26089531

[JEB249238C40] Meng, Y., Uchida, K., Sato, T., Yamamura, K. and Yamada, Y. (1999). Difference in the burst patterns of digastric and mylohyoid activities during feeding in the freely behaving rabbit. *Dysphagia* 14, 78-84. 10.1007/PL00009591 10028037

[JEB249238C41] Michna, H. (1989). Anatomical anomaly of human digastric muscles. *Acta Anat.* 134, 263-264. 10.1159/000146698 2728848

[JEB249238C42] Morgan, D. (1990). New insights into the behavior of muscle during active lengthening. *Biophys. J.* 57, 209-221. 10.1016/S0006-3495(90)82524-8 2317547 PMC1280663

[JEB249238C43] Muhl, Z. F. (1982). Active length–tension relation and the effect of muscle pinnation on fiber lengthening. *J. Morphol.* 173, 285-292. 10.1002/jmor.1051730305 7186549

[JEB249238C44] Muhl, Z. and Kotov, O. (1988). Muscle spindles in the digastric muscle of the rabbit. *J. Dent. Res.* 67, 1243-1245. 10.1177/00220345880670091801 2970484

[JEB249238C45] Muhl, Z. F. and Newton, J. H. (1982). Change of digastric muscle length in feeding rabbits. *J. Morphol.* 171, 151-157. 10.1002/jmor.1051710204 7062342

[JEB249238C46] Muhl, Z., Grimm, A. and Glick, P. (1978). Physiologic and histologic measurements of the rabbit digastric muscle. *Arch. Oral Biol.* 23, 1051-1059. 10.1016/0003-9969(78)90108-5 287421

[JEB249238C47] Orsbon, C. P., Gidmark, N. J. and Ross, C. F. (2018). Dynamic musculoskeletal functional morphology: integrating diceCT and XROMM. *Anat. Rec.* 301, 378-406. 10.1002/ar.23714 PMC5786282 29330951

[JEB249238C48] Orsbon, C. P., Gidmark, N. J., Gao, T. and Ross, C. F. (2020). XROMM and diceCT reveal a hydraulic mechanism of tongue base retraction in swallowing. *Sci. Rep.* 10, 8215. 10.1038/s41598-020-64935-z 32427836 PMC7237434

[JEB249238C49] Pataky, T. C. (2012). One-dimensional statistical parametric mapping in Python. *Comput. Methods Biomech. Biomed. Engin.* 15, 295-301. 10.1080/10255842.2010.527837 21756121

[JEB249238C50] Payne, R. C., Hutchinson, J. R., Robilliard, J. J., Smith, N. C. and Wilson, A. M. (2005). Functional specialisation of pelvic limb anatomy in horses (*Equus caballus*). *J. Anat.* 206, 557-574. 10.1111/j.1469-7580.2005.00420.x 15960766 PMC1571521

[JEB249238C51] Peck, D., Buxton, D. and Nitz, A. (1984). A comparison of spindle concentrations in large and small muscles acting in parallel combinations. *J. Morphol.* 180, 243-252. 10.1002/jmor.1051800307 6235379

[JEB249238C52] Percie Du Sert, N., Hurst, V., Ahluwalia, A., Alam, S., Avey, M. T., Baker, M., Browne, W. J., Clark, A., Cuthill, I. C. and Dirnagl, U. et al. (2020). The ARRIVE guidelines 2.0: updated guidelines for reporting animal research. *J. Cereb. Blood Flow Metab.* 40, 1769-1777. 10.1177/0271678X20943823 32663096 PMC7430098

[JEB249238C53] Roberts, T. J. and Gabaldón, A. M. (2008). Interpreting muscle function from EMG: lessons learned from direct measurements of muscle force. *Am. Zool.* 48, 312-320. 10.1093/icb/icn056 PMC4817590 21669793

[JEB249238C54] Ross, S. A., Waters-Banker, C., Sawatsky, A., Leonard, T. R. and Herzog, W. (2024). A methodological approach for collecting simultaneous measures of muscle, aponeurosis, and tendon behaviour during dynamic contractions. *Biol. Open* 13, bio060383. 10.1242/bio.060383 38780905 PMC11139038

[JEB249238C55] Schachner, E. R., Moore, A. J., Martinez, A., Diaz, R. E. Jr, Echols, M. S., Atterholt, J., Kissane, W. P. R., Hedrick, B. P. and Bates, K. T. (2024). The respiratory system influences flight mechanics in soaring birds. *Nature* 630, 671-676. 10.1038/s41586-024-07485-y 38867039

[JEB249238C56] Schwartz, G., Enomoto, S., Valiquette, C. and Lund, J. (1989). Mastication in the rabbit: a description of movement and muscle activity. *J. Neurophysiol.* 62, 273-287. 10.1152/jn.1989.62.1.273 2754478

[JEB249238C57] Tsuruyama, K., Scott, G., Widmer, C. and Lund, J. P. (2002). Evidence for functional partitioning of the rabbit digastric muscle. *Cells Tissues Organs* 170, 170-182. 10.1159/000046190 11731705

[JEB249238C66] Turnbull, W. D. (1970). Mammalian masticatory apparatus. Fieldiana (Geology) 18, 147-356.

[JEB249238C58] Van Eijden, T., Korfage, J. and Brugman, P. (1997). Architecture of the human jaw–closing and jaw–opening muscles. *Anat. Rec.* 248, 464-474. 10.1002/(sici)1097-0185(199707)248:3<464::aid-ar20>3.3.co;2-4 9214565

[JEB249238C59] Wakeling, J. M. and Johnston, I. A. (1999). White muscle strain in the common carp and red to white muscle gearing ratios in fish. *J. Exp. Biol.* 202, 521-528. 10.1242/jeb.202.5.521 9929455

[JEB249238C60] Watson, P. J., Gröning, F., Curtis, N., Fitton, L. C., Herrel, A., Mccormack, S. W. and Fagan, M. J. (2014). Masticatory biomechanics in the rabbit: a multi-body dynamics analysis. *J. R. Soc. Interface* 11, 20140564. 10.1098/rsif.2014.0564 25121650 PMC4233732

[JEB249238C61] Weijs, W. and Muhl, Z. (1987). The effects of digastric muscle tenotomy on jaw opening in the rabbit. *Arch. Oral Biol.* 32, 347-353. 10.1016/0003-9969(87)90090-2 3478037

[JEB249238C62] Wickiewicz, T. L., Roy, R. R., Powell, P. L. and Edgerton, V. R. (1983). Muscle architecture of the human lower limb. *Clin. Orthop. Relat. Res.* 179, 275-283. 10.1097/00003086-198310000-00042 6617027

[JEB249238C63] Widmalm, S. E., Lillie, J. and Ash, M. Jr (1988). Anatomical and electromyographic studies of the digastric muscle. *J. Oral Rehabil.* 15, 3-21. 10.1111/j.1365-2842.1988.tb00142.x 3162258

[JEB249238C64] Williamson, M. R., Dial, K. P. and Biewener, A. A. (2001). Pectoralis muscle performance during ascending and slow level flight in mallards (Anas platyrhynchos). *J. Exp. Biol.* 204, 495-507. 10.1242/jeb.204.3.495 11171301

[JEB249238C65] Xie, P., Jiang, Y., Zhang, X. and Yang, S. (2012). The study of intramuscular nerve distribution patterns and relative spindle abundance of the thenar and hypothenar muscles in human hand. *PLoS One* 7, e51538. 10.1371/journal.pone.0051538 23251569 PMC3519735

