# Supplementary material for: The functional role of the rabbit digastric muscle during mastication
Source: J Exp Biol. 2024 Sep 19;227(18):jeb249238. doi: 10.1242/jeb.249238 (PMC11449450; doi:10.1242/jeb.249238)
Supplement: Supplementary information [file jexbio-227-249238-s1.pdf]

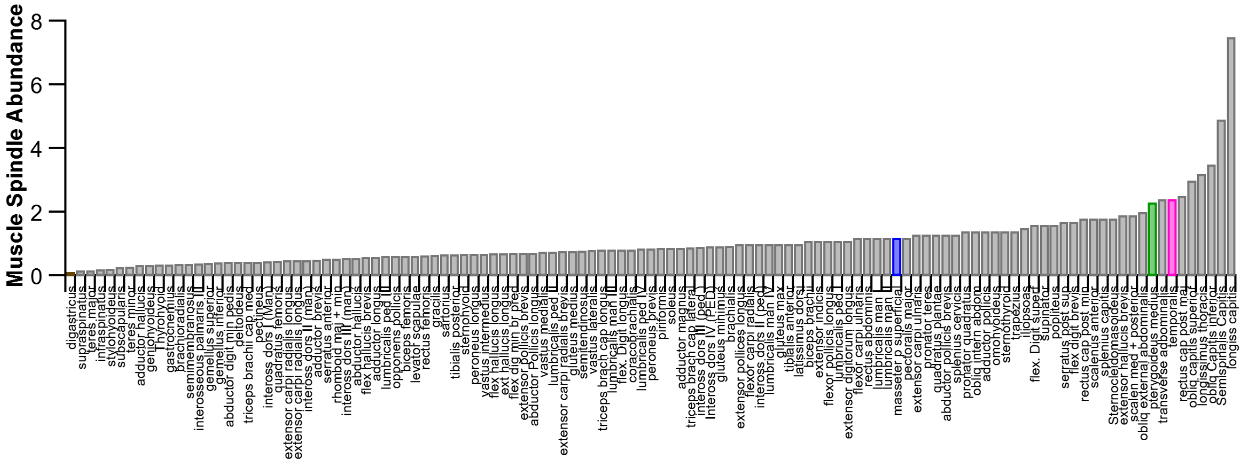

Fig. S1. Muscle spindle abundance across the human body. Muscle spindle abundance across 119 different human muscles taken from Banks (2006). The digastric (brown) has the lowest muscle spindle abundance across all muscles. Contrastingly, the superficial masseter (blue) medial pterygoid (green) and temporalis (pink) have some of the greatest abundance of muscle spindles across the body.

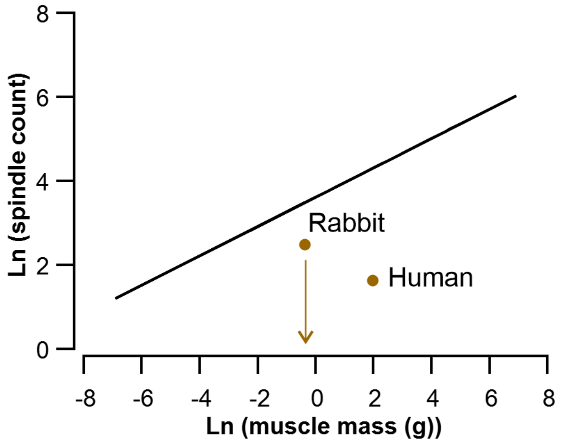

Fig. S2. Estimate of rabbit muscle spindle abundance. Homologous muscle equation line of best fit (Y=0.35X +3.62) taken from Banks (2006). Using muscle spindle counts from Muhl and Kotov (1988) we show that in the single example where an individual digastric muscle contained 12 spindles it sits bellow this line of best fit. This indicates that the rabbit digastric muscle has a low muscle spindle abundance for its given mass. It's likely that the rabbit digastric muscle sits even further bellow this line as 17 of the 18 digastric muscles examined by Muhl and Kotov (1988) presented with zero detectable muscle spindles.

Journal of Experimental Biology • Supplementary information

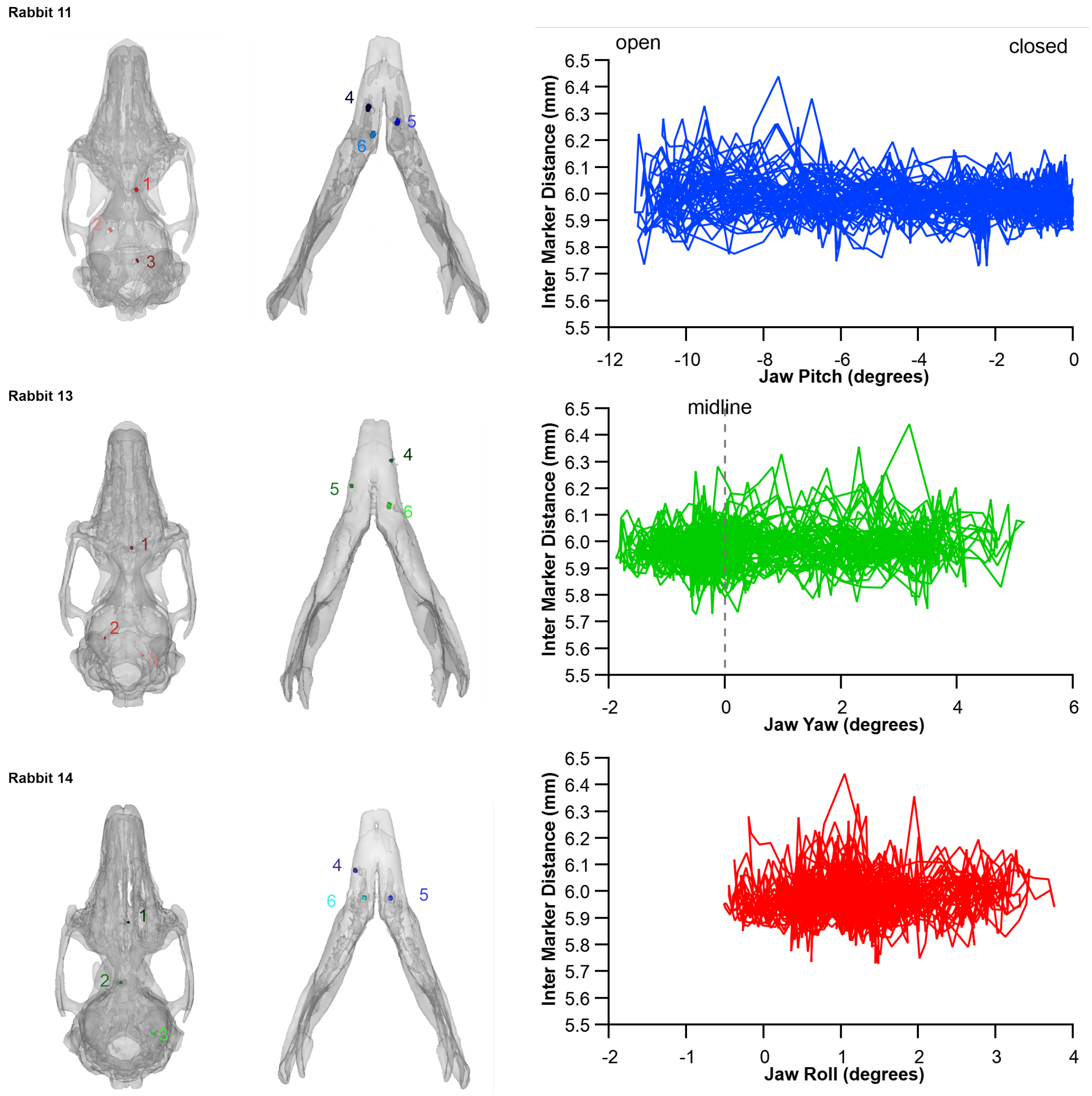

Fig. S3. Tantalum bead placement in the skull and mandible. 3 beads were placed in the skull and 3 in the mandible of each rabbit. Here the Inter-marker distance across the right (marker 5) and left (marker 6) hemimandible from Rabbit 11. The inter-marker distance across 30 consecutive masticatory cycles plot them against jaw pitch (blue) and jaw yaw (green), jaw roll (red). There appears to be no obvious relationship between the movement of the jaw and the inter-marker distance.

Journal of Experimental Biology • Supplementary information

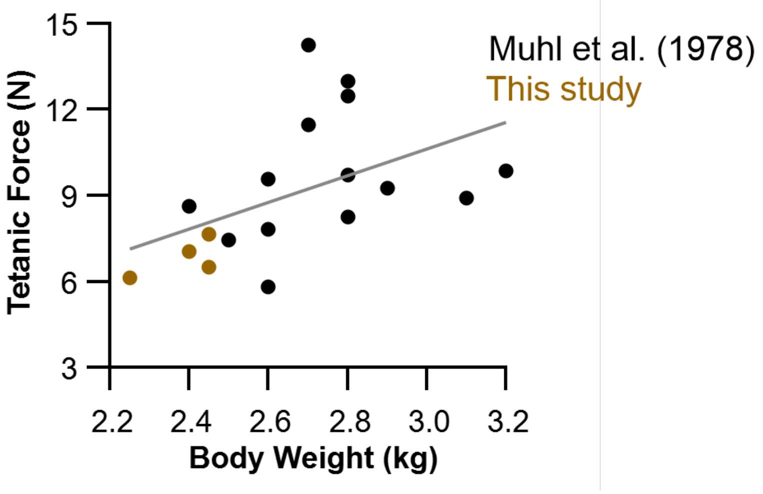

Fig. S4. The isometric tetanic force of the digastric muscle. Anapol, Muhl and Glick (1987) reported in the rabbit digastric muscle that body mass significantly correlated with tetanic force (P_0) ($r=0.62$, $P<0.001$), measured across 30 rabbits (body mass ranging 2.6-5.3kg). Here we have taken the raw data from 14 rabbits (black dots) from Table 1 in Muhl et al (1978) and combined our four rabbits from this study (brown dots). For the given mass of our rabbits, their forces are comparable to that of their body mass, therefore there cannot be any suggestion that our stimulation approach was sub maximally activating our muscles.

Journal of Experimental Biology • Supplementary information

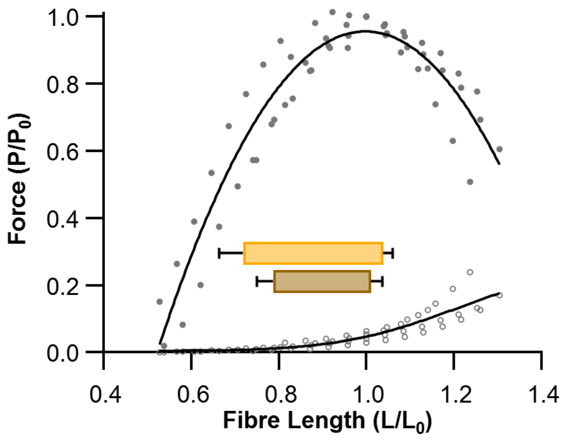

Fig. S5. The isometric twitch force-length properties and in situ operating lengths of the digastric muscle. Active muscle force (grey filled circles) and passive force (grey unfilled circles) relationship with estimated fibre length. Overlaid bar graphs depict the operating fibre lengths for the digastric muscle when functioning on the balancing (brown) and working (orange) side. Resting length in vivo was assumed to correspond to L_0. Note the small incremental increase in passive force with increasing length. Passive force at L_0 corresponds to 5.3% of maximum isometric twitch force, which equates to just 2% of isometric tetanic force.

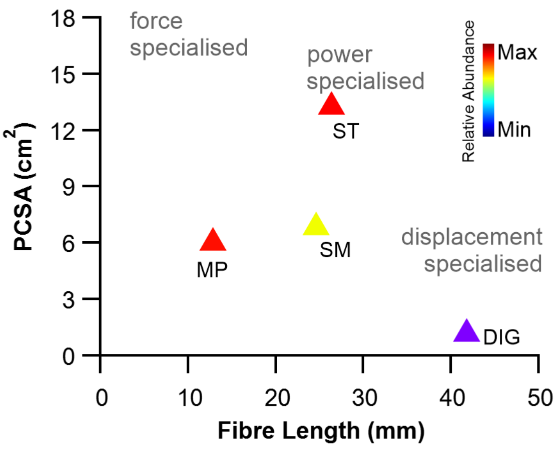

Fig. S6. Morphospace plot for the jaw muscles in humans. Fibre length and physiological cross-sectional area (PCSA) for human jaw muscles, plot with heat map of muscle spindle abundance. These data highlight that the digastric muscle is architecturally optimised to function as a displacement specialist compared to the major jaw closing muscles. These data do not support the hypothesis that muscle spindle abundance correlates with muscles optimised for displacement function and sensing (hypothesis 1). Medial pterygoid (PT), superficial masseter (SM), superficial temporalis (ST). The small PCSA and long fibre length of the DIG muscle suggests an architecture optimised to function as a displacement specialist.
